# Responses of spatial-temporal dynamics of bacterioplankton community to large-scale reservoir operation: a case study in the Three Gorges Reservoir, China

**DOI:** 10.1038/srep42469

**Published:** 2017-02-13

**Authors:** Zhe Li, Lunhui Lu, Jinsong Guo, Jixiang Yang, Jiachao Zhang, Bin He, Linlin Xu

**Affiliations:** 1 Key Laboratory of Reservoir Aquatic Environment of CAS, Chongqing Institute of Green and Intelligent Technology, Chinese Academy of Sciences, Chongqing 400714, China; 2 Key Laboratory of the Three Gorges Reservoir Region’s Eco-Environment, Ministry of Education, Chongqing University, Chongqing 400045, China; 3 College of Resources and Environment, Hunan Agricultural University, Changsha 410128, China

## Abstract

Large rivers are commonly regulated by damming, yet the effects of such disruption on bacterioplankton community structures have not been adequately studied. The aim of this study was to explore the biogeographical patterns present under dam regulation and to uncover the major drivers structuring bacterioplankton communities. Bacterioplankton assemblages in the Three Gorges Reservoir (TGR) were analyzed using Illumina Miseq sequencing by comparing seven sites located within the TGR before and after impoundment. This approach revealed ecological and spatial-temporal variations in bacterioplankton community composition along the longitudinal axis. The community was dynamic and dominated by Proteobacteria and Actinobacteria phyla, encompassing 39.26% and 37.14% of all sequences, respectively, followed by Bacteroidetes (8.67%) and Cyanobacteria (3.90%). The Shannon-Wiener index of the bacterioplankton community in the flood season (August) was generally higher than that in the impoundment season (November). Principal Component Analysis of the bacterioplankton community compositions showed separation between different seasons and sampling sites. Results of the relationship between bacterioplankton community compositions and environmental variables highlighted that ecological processes of element cycling and large dam disturbances are of prime importance in driving the assemblages of riverine bacterioplankton communities.

Prokaryotes are essential players in aquatic ecosystems, catalyzing significant biogeochemical reactions and playing central roles in aquatic food webs^1^,^2^. Natural assemblages of bacterioplankton are highly diverse and can undergo dynamics in community compositions in response to spatial-temporal environmental variation across ecosystems^3^. These fluctuations may result in changes in the functional roles of bacterial communities in the biogeochemical cycles^4^,^5^. There is reason to believe that bacterioplankton community indexes or indicator taxa could be developed, as a variety of studies have demonstrated that bacterioplankton have remarkable capabilities to respond to both natural and anthropogenic environmental changes^6^,^7^. However, many rivers are highly regulated for hydropower or water supply purposes, which cause critical hydrological disturbances at the spatial scale^8^. Reservoirs constitute a discontinuity for river systems, as they regulate water flow circulation, modify water residence time, and affect riverborne nutrients and material loads by retaining a large portion of suspended material transported by the river^9^,^10^. As a result, upstream and downstream sections of reservoirs tend to differ greatly in their physico-chemical properties^11^, and changes in the bacterioplankton communities are thus expected.

Seston sedimentation due to damming has been shown to cause shifts in the proportion of particle-attached bacteria^12^,^13^, which might imply changes in the composition and metabolic capabilities of the bacterial assemblages from both reaches^14^. Moreover, studies performed within reservoirs have reported large longitudinal shifts in bacterioplankton community structures that could ultimately be attributed to water residence time in the impoundment period^15^. Similarly, a clear differentiation in bacterioplankton assemblages was expected between upstream and downstream locations. However, the response of bacteria to environmental changes is not only due to replacement of the existing phylotypes but also to functional adjustments of the existing taxa^15^,^16^. Clearly, different bacterial assemblages were found in the small Sinnamary River before and after the reservoir, and large impoundments of the Danube River resulted in indiscernible effects on bacterial communities^17^,^18^. There are limited studies that compare bacterial communities before and after impoundments in reservoirs. Thus, the objective of this study was to determine the influence of damming on spatial and seasonal patterns of bacterial community compositions in a large regulated reservoir.

The Three Gorges Reservoir (TGR) was formed in 2003 after construction and impoundment of the Three Gorges Dam (TGD). The Three Gorges Reservoir is from Chongqing (west) to Yichang of Hubei province (east), with a distance of approximately 662.9 km^18^. The TGR, one of the largest reservoirs in the world, is comprised of a dammed river valley with a water surface area of up to 1,080 km^2^. According to its regulation plan, the water level in the TGR is impounded to 175 m for power generation during the winter and discharged to 145 m for flood control during the summer^8^. Thus, obvious aquatic ecosystem seasonal transitions are present within the TGR^19^, and operation of the TGD result in structurally distinctive limnological characteristics in the main stream of the Yangtze River^20^. The TGD along the Yangtze River causes disruptions to physicochemical variables in longitudinal profiles as well as algal biomass, species composition, diversity and richness^21^.

Most studies have focused on analyzing water quality variations and phytoplankton communities in TGR ecosystems^22^,^23^,^24^, yet have failed to analyze microbiology mechanisms within the TGR under regulation of the Three Gorges Dam. Bacterioplankton community structure compositions are likely to influence or be influenced by environmental factors (physico-chemical and hydrological) within the TGR ecosystems. Bacterioplankton community responses to environmental factors might highlight the susceptibility of reservoir microbes to environmental changes. Furthermore, the spatial-temporal variations in bacterioplankton community structures are potentially induced by the dynamic water level conditions imposed by hydropower operations of the TGD. Therefore, it is important to analyze both spatial-temporal variations of bacterioplankton community compositions in the entire TGR and to estimate whether changing environmental factors are responsible for driving bacterioplankton community structure distributions.

In this study, 16 S rRNA gene high-throughput sequencing was conducted in different areas of the reservoir along the longitudinal axis of the TGR in order to gain in-depth insight into possible environmental factors shaping spatial-temporal variations in bacterioplankton community structures. This knowledge can also help to better understand specific bacterioplankton roles in biogeochemical cycling within the TGR and determine the potential effects of river regulations due to damming on bacterioplankton communities.

## Results

### Environmental conditions and physiochemical characteristics within the TGR

**Figure 1:**
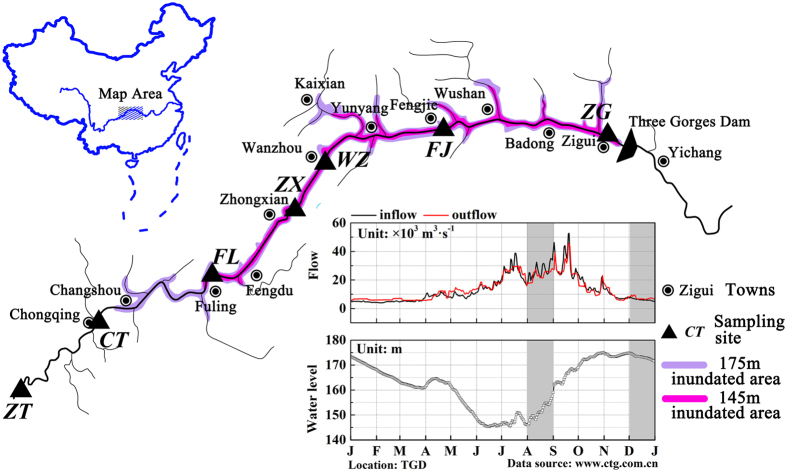
Maps of the Three Gorges Reservoir showing the location of the sampling sites. Base map of the Yangtze river systems in the Three Gorges Reservoir and map of China were created by Adobe Phososhop CS5. Sub-graph of daily variations of reservoir in- and outflows, as well as reservoir water levels, was generated by OriginLab 9.4. Data source was from the web site of the China Three Gorges Cooporation (www.ctg.com.cn). The whole figure was combined by Adobe Phososhop CS5.

Environmental conditions and physiochemical characteristics of the samples from the seven investigated areas (Fig. 1) are shown in Table 1. The distances of the sampling sites from the TGD are shown in Table 2. Samples collected during the flood season (August 2014) were generally characterized by higher water temperature, conductivity, total phosphorus (TP) and total nitrogen (TN). TN content ranged from 1.73 to 3.23 during the flood season and from 1.44 to 1.84 during the dry season. Moreover, TN and total organic carbon (TOC) concentrations at downstream locations of sites Wanzhou (WZ), Fengjie (FJ) and Zigui (ZG) were higher those at the upstream locations of Zhutuo (ZT), Fuling (FL) and Cuntan (CT). However, dissolved oxygen (DO), total carbon (TC) and TOC concentrations in the flood season were lower than in the dry season. TC content ranged from 48.39 to 54.74 mg/L in the flood season and from 56.50 to 66.37 mg/L in the dry season. Additionally, TOC content ranged from 3.82 to 5.79 mg/L in the flood season and from 5.33 to 8.11 mg/L in the dry season. Water temperatures in downstream sections of the reservoirs were 2–5 °C higher than upstream sites.

### Chlorophyll a (Chl a) concentrations within the TGR

**Figure 2:**
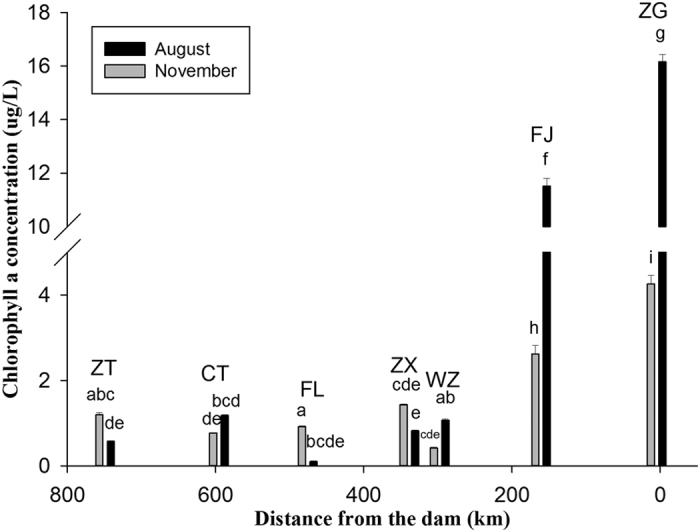
Variation of chlorophyll *a* concentration in the Three Gorges Reservoir. Axis X represents distance of the sampling sites (ZT, CT, FL, ZX, WZ, FJ, and ZG, in order) from the TGD. The distances of the sampling sites from the dam were 749.25, 595.54, 475.68, 338.48, 297.81, 160.91, and 4.74 km, in order.

The temporal variations in Chl *a* concentrations at reservoir sampling sites highlighted seasonal and locational differences (Fig. 2). Chl *a* concentrations in the flood season (August 2014) were generally higher than concentrations in the dry season (November 2014). It is worth noting that Chl *a* concentrations at downstream sites (ZG and FJ) of the TGR (averaged 13.84 mg/L in flood season and 3.44 mg/L in dry season) were higher than concentrations at upstream sites (WZ, ZX, FL, CT and ZT, with an average of 0.75 mg/L and 0.95 mg/L in flood and dry seasons, respectively). Chl *a* concentrations at ZG, near the dam, reached 16.16 mg/L in the flood season, indicating eutrophication in the reservoir. This is in accordance with previous studies that also showed an increase in Chl *a* concentrations downstream from the reservoir^20^.

### Bacterioplankton community composition within the TGR as determined by sequencing and taxa identification

**Figure 3:**
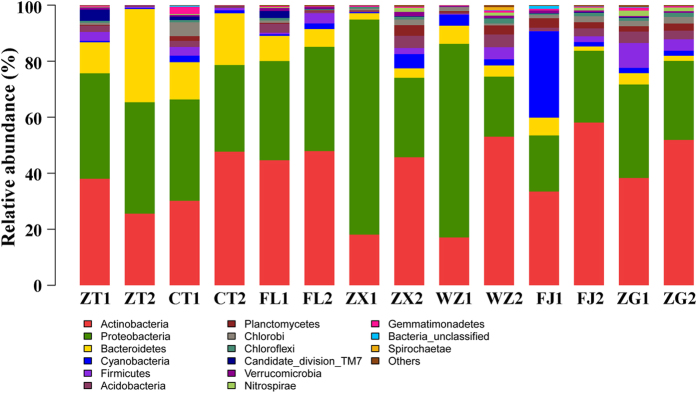
The relative percent contribution of the bacterioplankton community composition at the phyla level for the different sample sites. The phylogenetic groups account for < 1% of all quality sequences are grouped together in “others”.

Upon chimera removal, a total of 230,202 sequences were obtained from Illumina Miseq sequencing, all samples were rarefied at 9,250 sequences per sample to retain as many samples as possible. The Good’s estimator of coverage was > 98.5% for all samples. The RDP classifier^25^ and Greengene database^26^ were used to assign taxonomy to OTUs from domain to genus levels. OTUs were used to calculate Shannon-Wiener diversity and richness indexes, and these data are shown in Table 3. A total of 715 OTUs were identified and assigned into 310 genera, 190 families, 109 orders, 57 classes and 26 phyla. For whole gene sequences obtained from each sample, rarefaction curves were established at a nucleotide genetic distance of 0.03 (Figure S1). An analysis of OTUs showed variability along the longitudinal axis with water level changes within the TGR. At the phylum level, a total of 26 different bacterial phyla were identified in each sample. Across all sites, bacterioplankton communities were dominated by Proteobacteria and Actinobacteria, and these two phyla accounted for 39.26% and 37.14% of all sequences, respectively. Bacteroidetes (8.67%) and Cyanobacteria (3.90%) were also present in the remaining sequences. Only 0.15% of sequences could not be classified at the phylum level, and they were defined as bacteria-unclassified. The bacterioplankton community composition at the phylum level in different samples is shown in Fig. 3. The most common classes were Betaproteobacteria (19.09%), Acidimicrobia (11.61%) and Alphaproteobacteria (10.99%). The most abundant genera were *hgcI_clade* (15.50%), *Limnohabitans* (7.15%) and Sporichthyaceae_unclassified (4.19%).

### Spatial-temporal variations of bacterioplankton community composition within the TGR

The diversity and richness of each sample based on OTU analysis was assessed by calculating the Shannon-Wiener index (H), Simpson Index, Chao and ACE richness estimators (Table 3). The Shannon-Wiener indices of the bacterioplankton community in the flood season (August, 2014) were generally higher than those in the dry season (November, 2014) except at sampling sites ZX and FJ. The observed OTU numbers and Shannon-Wiener index at sampling site ZT (furthest from the TGD) in November were the lowest (238 and 3.45, respectively).

**Figure 4:**
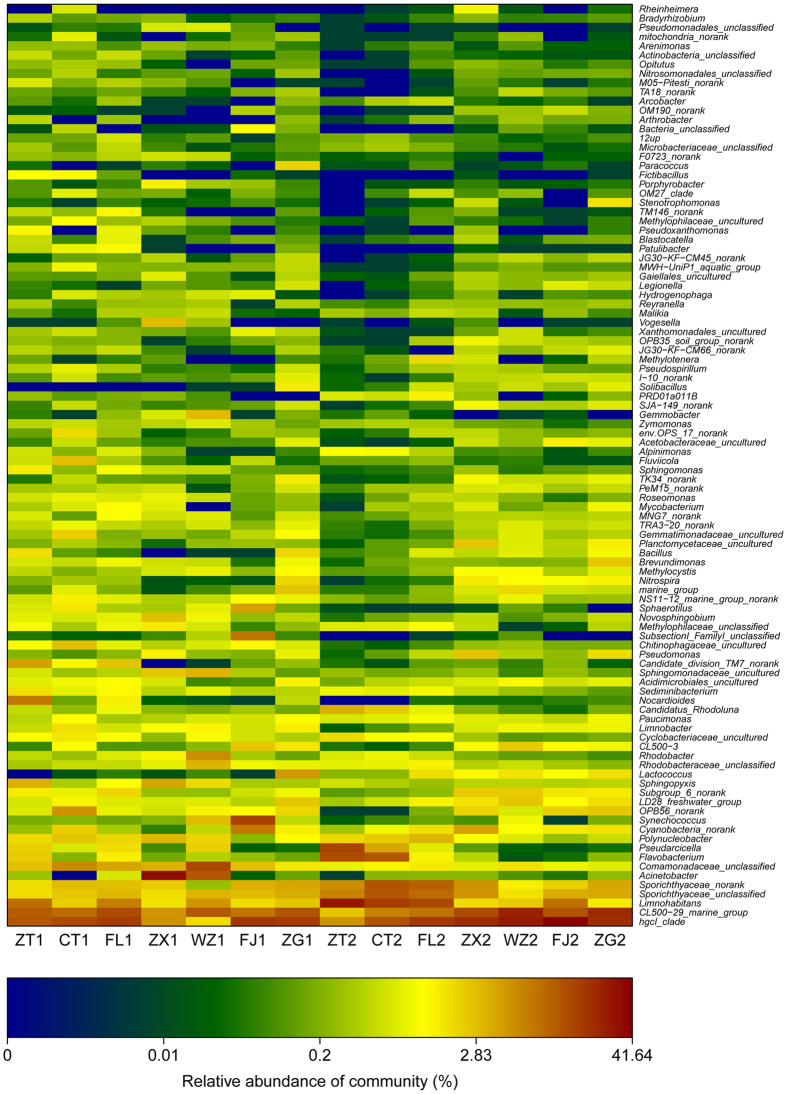
Heatmap of the relati ve abundances of the top 100 genera of bacterioplankton among the different sampling times and sites. Samples cluster according to hierachcial clustering based on Bray-Curtis distance calculated from the phylotype relative abundance. The heat map was generated with R version 3.0.1 using the vegan package.

**Figure 5:**
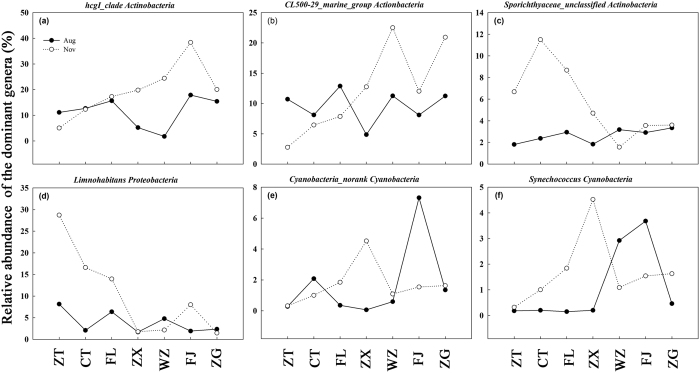
Temporal and longitudinal dynamics of the relative abundance of the dominant genera. Axis X represents distance of the sampling sites (ZT, CT, FL, ZX, WZ, FJ, and ZG, in order) from the Three Gorges Dam (TGD). Solid and hollow circles representing the samples collected in August and November 2014, respectively. The figure was generated with Sigmaplot version 12.5.

The bacterioplankton community structure was analyzed at the genus level using a heat map of the top 100 genera for the different sites and times (Fig. 4). Spatial-temporal variations of the six most abundant genera were analyzed along the longitudinal axis within the TGR. Of the six most abundant genera, three belonged to Actinobacteria, one to Proteobacteria and two to Cyanobacteria (Fig. 5). The relative abundance of the six genera were, for the most part, higher in November than in August.

**Figure 6:**
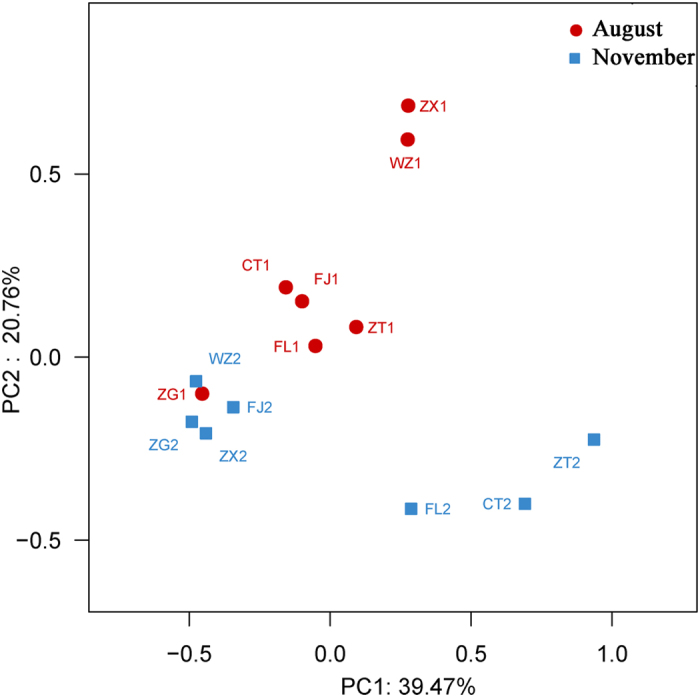
Principal coordinate analysis (PCoA) of bacterioplankton community based on the Bray-Curtis similarity between samples. Percentage of the variation between the samples explained by each axis is indicated on the figure. Red circles and blue squares represent the samples collected in August and November 2014, respectively.

Statistical analysis of the OTU distribution of bacterioplankton community composition along the longitudinal axis of the impoundment during the dry season was performed by principal coordinate analysis (PCoA). PCoA of the bacterial community compositions yielded a relatively good separation between different seasons (impoundment season and dry season) and positions (upstream and downstream), which can be seen in Fig. 6. Principal components 1 and 2 accounted for 56.19% of the total sample variability (39.47% and 20.76% for PC1 and PC2, respectively). PcoA coordinates revealed a grouping of the samples into flood season (ZT1, CT1, FL1, ZX1, WZ1, FJ1 and ZG1) and dry season clusters (ZT2, CT2, FL2, ZX2, WZ2, FJ2 and ZG2), which can be explained by their similar compositions of primary species. Moreover, PCoA plots showed comparatively high discrepancies between upstream and downstream sites, such as ZT2 grouped closer together with CT2 and FL2, suggesting a high similarity of bacterioplankton communities at these three sites.

### Distribution of bacterioplankton community assemblages in relation to environmental variables within the TGR

**Figure 7:**
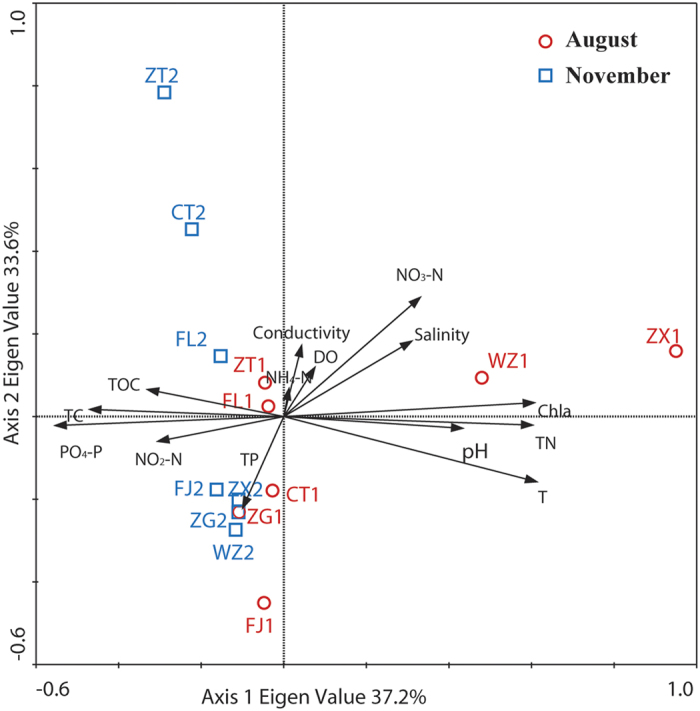
Redundancy analysis of bacterioplankton community compositions and environmental variables. Circles and squares represent the samples collected in August and November 2014, respectively.

To further determine the environmental variables associated with changes in bacterioplankton community structure, redundancy analysis (RDA) was performed with contextual parameters that significantly explained variations in bacterioplankton fingerprints. The first two axes explained up to 37.2% of RDA 1 and 33.6% of RDA 2 of the total variation in bacterial community structure (Fig. 7). The environmental variables, referenced in Table 1, were comprised of DO, TC, Chl *a*, pH, conductivity, phosphate phosphorus (PO_4_-P), nitrite nitrogen (NO_2_-N), nitrate nitrogen (NO_3_-N), ammonium nitrogen (NH_4_-N), salinity, water pH and temperature. Of all the environmental factors investigated, Chl *a* appeared to have the most significant influence on bacterioplankton community assemblages. Most variances can be explained by water temperature, Chl *a*, PO_4_-P, TC and TN. There is a positive association of Axis 1 with water temperature, pH, Chl *a* and TN and a negative association of Axis 1 with TC, TOC, PO_4_-P and NO_2_-N. Water temperature, pH, Chl *a* and TN explained 13.5%, 12.8%, 11.3%, 9.8% and 7.2% of the variation in the bacterioplankton community, respectively. The partial RDA model statistically explained 38.6% of the variation (P < 0.05) in the bacterioplankton community composition (Table 4). The variation shared by significant factors was 16.0%.

## Discussion

It has been well documented that river regulations due to damming would affect water physico-chemical conditions, sediment transportation^27^, phytoplankton compositions^15^,^22^, and bacterioplankton communities^19^ in the reservoirs. However, very few studies have focused on bacterioplankton community structures based on Miseq sequencing within the TGR. This study may be among the first to investigate and evaluate the potential effects of river regulations due to damming on bacterioplankton community composition along the longitudinal axis of the TGR.

### Effects of reservoirs on water physico-chemical conditions

Reservoirs exhibit a marked degree of spatial heterogeneity in phytoplankton productivity and biomass as a result of the longitudinal gradient in the basin morphology, flow velocity, water residence time, suspended solids, and light and nutrient availability^28^. The TGR is a typical reservoir containing three typical zones that are distinguishable along the longitudinal axis (riverine zone, transition zone and lacustrine zone). The riverine zone consists of the ZT, CT and FL sites, the transition zone consists of the ZX and WZ sites, and the lacustrine zone consists of the FJ and ZG sites near the TGD. There are inherent differences between the upstream and downstream sites within the TGR. The upstream riverine zone (ZT, CT and FL) is a lotic environment that is characterized by lower conductivity and lower concentrations of NO_3_-N, TOC, particulate matter, DOC and Chl *a* relative to the downstream sites (Table 1, Fig. 2). These findings have been previously reported in the TGR^29^. The lacustrine zone (FJ and ZG) is characterized by higher Chl *a* (Fig. 2) that was probably due to the higher phytoplankton productivity and biomass that occurs in conjunction with the decreasing flow velocity at these two sites^24^. The decreasing flow velocity at these two sites increases nutrient retention downstream and homogenizes water temperatures, which favors phytoplankton reproduction^30^.

Anthropogenic activities might attribute to the different characteristics of the upstream and downstream sites. For example, along the longitudinal axis, sites CT, FL and WZ were near a large metropolitan area with a large population and sizeable industries, including natural gas chemical industries, petrochemical industries, new energy resources, and steel and equipment manufacturing^31^. These industries are the main source of new chemical materials and the heavy chemical industry in Chongqing City, which has resulted in more external nutrients flowing into the TGR^31^,^32^. As such, the NO_3_^−^-N, TP, TN, and TOC contents in these three monitoring sections were generally higher than those in other sections.

Miseq sequencing results based on 16 S rRNA indicated that there was higher α-diversity of bacterioplankton in August than in November (Table 3). This is in accordance with a previous study^33^. In another study, nutrient levels were considered one of the main factors in controlling seasonal variations in bacterial communities^34^. The Yangtze River is highly regulated for hydropower and water supply purposes. The water level in the TGR is impounded to 175 m for power generation during the winter and discharged to 145 m for flood control during the summer. These water level changes have generated significant hydrological disturbances along the longitudinal axis.

Figure 6 shows that samples were separated by site and month, suggesting that bacterioplankton exhibit complex and sensitive responses to different environments. Samples ZT2, CT2 and FL2 were grouped together, suggesting that bacterioplankton community compositions in the riverine zone of the TGR have similar characteristics in November. A succession in the composition of the bacterioplankton community along the longitudinal axis from upstream to downstream was observed (Fig. 3). It was hypothesized that the structural and functional patterns of communities was potentially impacted by TGD regulations. Water flow velocity, which is one of the most important factors affecting plankton in river systems, can be significantly decreased by damming regulation^35^. Seven representative sites along the TGR area represent direct dam effects at different sites.

### Effects of reservoirs on bacterioplankton community compositions

The relatively higher abundance of *hgcI_clade* and *Limnohabitans* was in accordance with previous research^36^. The most abundant genus, the *hgcI_clade* (Fig. 5), is affiliated with Actinobacteria and is common in a wide range of freshwater habitats. A recent single-cell genomic study showed that this clade had a strong genetic ability to take N-rich and carbohydrate organic compounds^37^. The *hgcI_clade* also has the potential to use sunlight through the actinorhodopsin gene^38^, which may promote anaplerotic carbon fixation, indicating both autotrophic and heterotrophic lifestyles of this clade^37^. Another abundant genus, *Limnohabitans* belongs to the Betaproteobacteria. It has been suggested that *Limnohabitans* plays an important role in water bacterioplankton communities because of its high rates of substrate uptake and growth on algae-derived substrates^39^. Previous studies have shown that *Polynucleobacter* and *CL500-29_marine_group* were driven by NO_3_^−^/NO_2_^− ^40,41. However, the nitrification capability of the hypoxia-adapted *Marine Group* is unknown and is worth further investigation. It is interesting that *Pseudarcicella* and *Flavobacterium* within the Bacteroidetes phylum were predominantly found at site ZT in November (Fig. 4). The high turbidity in the bottom water would prevent the penetration of light to the bottom and thus inhibit the growth of *Synechococcus* and *Cyanobacteria_norank* (both belong to Cyanobacteria phylum) at sites ZT and CT. As mentioned above, high nutrient loads might favor the growth of these specific groups, and this suggests that there may be anthropogenic activity resulting in higher nutrient zones at these sites. High nutrient concentrations resulted in high Chl *a* concentrations of X at sites FJ and ZG, and this is consistent with the higher Cyanobacterial biomass at these two sites^42^.

### Bacterioplankton community compositions in relation to environmental variables

To determine whether water hydrography and environmental factors have on impact on bacterioplankton community composition, redundancy analysis was performed on X and Y datasets (Fig. 7). Water temperature, Chl *a*, PO_4_-P, TC and TN best explained the observed variability in bacterioplankton community composition. The result was in accordance with several studies that confirmed that the phytoplankton community and water temperature were influential factors on the seasonal variation of bacterioplankton community composition^43^,^44^. Water temperature is always a potentially limiting factor because microbes have optimum temperature characteristics^45^. Thus, bacterioplankton community composition changes as the water temperature fluctuates. In this study, Chl *a* was also a significant factor on bacterioplankton community composition. Concentration of Chl *a* was mainly associated with the biomass of phytoplankton^46^. It has been reported that phytoplankton plays an important role in regulating bacterioplankton community composition in natural systems^47^,^48^. The reason for this phenomenon was probably that the dissolved organic matter produced by the phytoplankton was considered an important carbon source for the bacteria^49^,^50^.

In conclusion, this study investigated bacterioplankton community assemblages and specific taxa in the large-scale Three Gorges Reservoir ecosystem using high-throughput sequencing. There were differences in bacterioplankton community structures along the longitudinal axis of the TGR and between the two operational periods (August and November) that show clear shifts in bacterial clades within the same taxa. Our results showed that bacterioplankton community assemblages in the TGR were mostly related to spatial parameters rather than physical-chemical variables. This suggests that ecological processes pertaining to river network length are of prime importance.

## Methods

### Study sites and sample collection

Seven sampling locations along the longitudinal axis within the TGR were selected from upstream to downstream: Zhutuo (ZT), Cutan (CT), Fuling (FL), Zhongxian (ZX), Wanzhou (WZ), Fengjie (FJ) and Zigui (ZG, near the TGD) (Fig. 1). Sampling sites were selected to span the entirety of the TGR within the Yangtze River. According to the reservoir’s operation strategy, the water level in the TGR is impounded to 175 m for power generation at the end of October. Water level is maintained during the whole dry season mainly for hydropower production. In February of the coming year, water level in the TGR is gradually decreases for maintaining downstream flow and preparing reservoir capacity for the incoming floods until the end of May. The water level is maintained at 145 m before the flood season^19^,^51^. The frequency distribution of water residence time of the TGR in 2014 (Figure S2), calculated based on daily data of reservoir capacity and reservoir inflow, showed that there were generally two to three peaks of counts in daily water residence time in the reservoir. Water residence time in August and November covers two of them, representing the two states of the reservoir, i.e., the lotic and lentic, throughout the year. In this case, samples were collected in August and November (2014) to capture the variations of bacterioplankton community potentially resulting from the impoundment and discharge stages of the TGD.

At each site, water samples (5 L) were collected from 0.5, 5 and 10 m of the middle point of the river and mixed fully in a bucket. The water samples were then subsampled for DNA-based analysis and physiochemical analyses. One liter of sample water was subsequently filtered on board through a 0.22-μm filter paper (Millipore, MA, USA) for bacterial DNA. Upon return, the filters were stored at −86 °C until DNA extraction. Water samples were also pre-processed based on standard methods for physicochemical analysis.

### Physicochemical analyses

Water stage data was collected from the monitoring department of the China Three Gorges Corporation (http://www.ctgpc.com.cn/). Temperature, dissolved oxygen (DO), pH and conductivity were measured using a YSI Pro2030 (YSI Incorporated, Ohio, USA) *in situ*. TC, TOC and TN were measured by a Vario TOC Cube analyzer (Elemen-tar, Hanau, Germany). NH_4_-N, NO_3_-N, NO_2_-N, TP, and PO_4_-P were measured with a UV-2700 spectrophotometer (Shimadzu, Kyoto, Japan) using a colorimetric method as described previously^29^. Chl *a* was extracted from a Whatman GF/C filter for 24 h with 90% acetone, centrifuged at 3,000 rpm for 10 min and then spectrophotometrically quantified.

### DNA extraction and Polymerase Chain Reaction (PCR)

Total DNA was extracted from the 0.22-μm filters using a PowerWater DNA Extraction kit (Mo Bio, CA, USA) according to the manufacturer’s instructions. The V3-V4 region of bacterial 16 S rRNA gene was amplified using primer set 338 F/806R^52^. All PCR amplifications were conducted in triplicate for each sample.

### Illumina MiSeq sequencing and analysis

Amplicons were gel purified using the AxyPrep DNA Gel Extraction Kit (Axygen Biosciences, CA, USA) according to the manufacturer’s instructions and quantified using QuantiFluor™-ST (Promega, USA). Purified amplicons were pooled in equal amounts and paired-end sequenced on the Illumina MiSeq platform at a read length of 2 × 300 bp (Majorbio, Shanghai)

Trimmed sequences were paired-end aligned using fastq-join and screened for quality using the following parameters: quality score ≥ 35 over a 50 nt window, no ambiguous bases, homopolymers ≤ 8 nt, and primer and barcode matching with 100% identity^53^. Sequences were chimera checked using UCHIME software^54^. The operational taxonomic units (OTUs) were picked at 97% sequence similarity and identified using the Ribosomal Database Project (RDP) classifier retrained with Greengenes (http://greengenes.secondgenomes). The NAST algorithm was used for alignment and Greengenes. Sequences were quality filtered and rarefied to 9,725 sequences per sample. OTU based analyses [sequencing coverage estimation, the Shannon-Wiener index (*H*), Chao and ACE richness estimators] were performed using the Quantitative Insights Into Microbial Ecology (QIIME) program^55^.

### Statistical analyses and graphical outputs

The three datasets generated by Chl *a* concentration were performed using SPSS Statistics software v. 19.0 (IBM, Armonk, NY) by One-way analysis of variance (ANOVA) followed by Tukey’s post hoc test^56^.

To give further insight into bacterioplankton community composition and environmental variable associations, RDA was used by means of the varpart function in the VEGAN R package^57^. Forward-selected environmental variables were confirmed as *P* < 0.05 in the selection procedure by Monte Carlo permutation test. Moreover, to differentiate the sole influence of each significant parameter, partial RDA was performed following the forward selection.

### Nucleotide sequence accession numbers

The sequences obtained from Miseq sequencing were submitted to the National Center for Biotechnology Information Sequence Read Archive database (http://www.ncbi.nlm.nih.gov/sra/) under the BioProject accession number PRJNA305461.

## Additional Information

**How to cite this article:** Li, Z. *et al*. Responses of spatial-temporal dynamics of bacterioplankton community to large-scale reservoir operation: a case study in the Three Georges Reservoir, China. *Sci. Rep.* **7**, 42469; doi: 10.1038/srep42469 (2017).

**Publisher's note:** Springer Nature remains neutral with regard to jurisdictional claims in published maps and institutional affiliations.

## Supplementary Material

Supplement Materials

## Figures and Tables

**Table 1:** Summary of the physiochemical characteristics for each of the sample sites taken during August and November 2014.

Sampling Time	Sampling Site	T^a^ (°C)	DO^b^ (mg/L)	pH	Salinity (ppt)	Conductivity (μS/cm)	TP^c^ (mg/L)	PO_4_-P (mg/L)	NO_3_-N (mg/L)	NO_2_-N (μg/L)	NH_4_-N (μg /L)	TC^d^ (mg/L)	TOC^e^ (mg/L)	TN^f^ (mg/L)
**August 2014**	***ZT***	22.60	8.86	8.02	0.10	305.9	0.11	0.032	0.67	0.97	0.80	53.83	5.13	1.73
	***CT***	23.40	8.44	8.06	0.20	315.2	0.16	0.052	0.80	1.25	4.45	53.54	3.82	1.84
	***FL***	23.00	8.41	7.90	0.20	322.9	0.18	0.037	0.25	1.75	2.22	54.22	4.24	3.23
	***ZX***	26.50	7.46	7.81	0.20	323.3	0.18	0.063	2.00	0.69	0.13	48.39	5.55	2. 40
	***WZ***	26.20	7.27	7.71	0.20	354.4	0.20	0.059	1.49	1.30	0.25	50.84	5.79	2.33
	***FJ***	27.50	7.15	7.52	0.20	350.5	0.12	0.029	1.69	0.65	2.42	54.74	4.72	2.22
	***ZG***	27.30	9.68	8.12	0.20	327.7	0.073	0.0077	1.69	0.13	6.46	54.40	4.57	2.81
**November 2014**	***ZT***	13.70	9.91	7.79	0.20	312.5	0.078	0.054	1.18	1.79	3.60	56.50	5.57	1.44
	***CT***	12.90	9.86	8.02	0.10	217.2	0.086	0.066	1.36	2.33	4.27	64.40	5.93	1.57
	***FL***	13.16	9.40	7.54	0.20	411.4	0.16	0.095	1.47	2.50	4.63	62.60	6.24	1.64
	***ZX***	15.91	8.30	6.90	0.16	330.8	0.11	0.093	0.82	0.13	0.86	66.37	5.33	1.57
	***WZ***	16.47	8.19	7.56	0.15	320.0	0.13	0.10	1.61	0.13	2.20	61.73	8.11	1.84
	***FJ***	16.83	8.05	7.55	0.15	322.6	0.12	0.080	1.53	0.01	1.47	61.64	6.31	1.62
	***ZG***	17.78	8.83	7.83	0.15	313.0	0.10	0.056	1.49	0.31	5.73	61.67	7.23	1.68

Abbreviations: a temperature; b Dissolved oxygen; c Total phosphorus; d Total carbon; e total organic carbon; f Total nitrogen. Seven sampling locations along the the longitudinal axis in TGR were selected from upstream to downstream: riverine zone consisted of sites Zhutuo (ZT), Cutan (CT), Fuling (FL); the transition zone consisted of sites Zhongxian (ZX), Wanzhou (WZ); the lacustrine zone consisted of Fengjie (FJ) and Zigui (ZG).

**Table 2:** The distance of the sampling sites from the Three Gorges Dam.

Sampling Site	ZT	CT	FL	ZX	WZ	FJ	ZG
Distance from the dam (km)	749.25	595.54	475.68	338.48	297.81	160.91	4.74

Abbreviations: The full name of each sampling site is listed in Table 1.

**Table 3:** Table showing the Operational Taxonomic Units (OTUs) for each sample site in August and November.

Sampling Time	Sampling Time Site	Sampling OTU	Ace	Chao	Coverage	Shannon	Simpson
**August 2014**	***ZT***	423	486	492	0.9906	4.71	0.0193
	***CT***	435	504	502	0.9908	5.03	0.0125
	***FL***	458	518	521	0.9905	4.87	0.0174
	***ZX***	343	418	417	0.9911	4.11	0.058
	***WZ***	332	406	417	0.9910	4.30	0.0299
	***FJ***	351	431	432	0.9906	4.10	0.0422
	***ZG***	475	537	568	0.9901	4.93	0.0159
**November 2014**	***ZT***	238	413	377	0.9906	3.45	0.0643
	***CT***	310	472	394	0.9897	3.88	0.0453
	***FL***	377	456	458	0.9898	4.24	0.035
	***ZX***	483	576	602	0.9876	4.71	0.0241
	***WZ***	416	482	500	0.9902	4.42	0.0385
	***FJ***	415	502	507	0.9889	3.98	0.0644
	***ZG***	433	488	489	0.9910	4.37	0.043

Richness estimators (Chao and Ace) and Diversity indicles (Shannon and Simpson) of the bacterial 16 S rRNA for each sample site were determined using OTU based analyses. Abbreviations: The full name of each sampling site is listed in Table 1.

**Table 4:** Partial redundancy analysis results of the influence of each significant parameter on the bacterioplankton community composition.

Parameters included in the model	Eigen value	Variation explained solely (%)	*P* value	Pseudo-*F* value
Water temperature	0.135	13.5	0.003	2.04
Chl *a*	0.128	12.8	0.009	1.48
PO_4_-P	0.113	11.3	0.015	1.51
TC	0.098	9.8	0.021	1.45
TN	0.072	7.2	0.024	1.18
All the above parameters together	0.386	38.6	0.016	2.64

Partial RDAs were based on the Monte Carlo test (n = 499) kept only the significant factors in the models. For each partial model, the other significant factors were used as covaribles.
