# Supplementary material for: Responses of spatial-temporal dynamics of bacterioplankton community to large-scale reservoir operation: a case study in the Three Gorges Reservoir, China
Source: Sci Rep. 2017 Feb 13;7:42469. doi: 10.1038/srep42469 (PMC5304162; doi:10.1038/srep42469)
Supplement: Supplement Materials [file srep42469-s1.pdf]

Supplementary information

Responses of spatial-temporal dynamics of bacterioplankton community to large-scale reservoir operation: a case study in the Three Georges Reservoir, China

Zhe Li[1], Lunhui Lu[1], Jinsong Guo[1,2], Jixiang Yang[1], Jiachao Zhang[3], Bin He[2], Linlin Xu[2]

[1]Key Laboratory of Reservoir Aquatic Environment of CAS, Chongqing Institute of Green and Intelligent Technology, Chinese Academy of Sciences, Chongqing 400714, China; [2]Key Laboratory of the Three Gorges Reservoir Region's Eco-Environment, Ministry of Education, Chongqing University, Chongqing 400045, China; [3]College of Resources and Environment, Hunan Agricultural University, Changsha 410128, China

Correspondence: L. Lu and J. Guo, Key Laboratory of Reservoir Aquatic Environment of CAS, Chongqing Institute of Green and Intelligent Technology, Chinese Academy of Sciences, Chongqing 400714, China.

E-mail: lulunhui@cigit.ac.cn and guojs@cigit.ac.cn

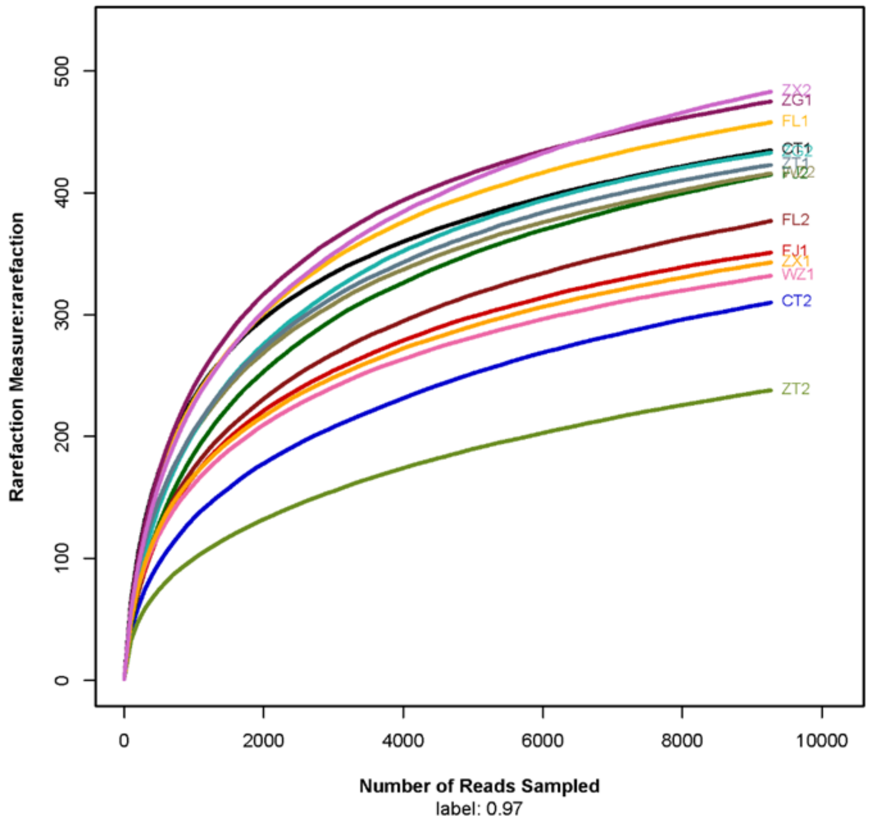

Figure S1 Rarefaction curve for bacterioplankton 16S rRNA gene fragments of the 14 samples. Operational taxonomic units (OTUs) were formed at genetic distances of 3%.

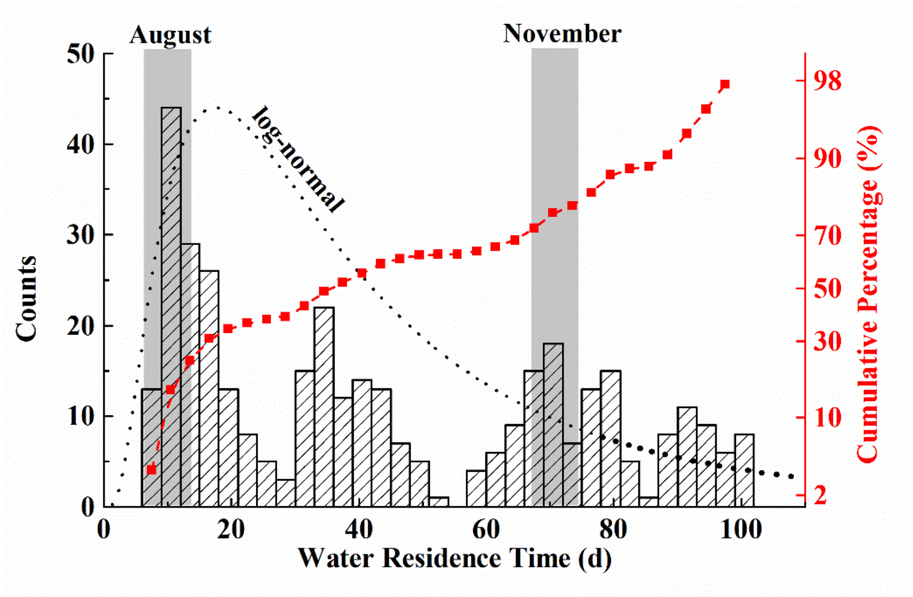

Figure S2 Frequency distribution of water residence time of the Three Gorges Reservoir in 2014. Black dots is the log-normal distribution curve while the red diamonds line shows the cumulative percentage of the samples. Dark grey area showed the water residence time of August and November in 2014.
